# Diversity and altitudinal distribution of Chrysomelidae (Coleoptera) in Peregrina Canyon, Tamaulipas, Mexico

**DOI:** 10.3897/zookeys.417.7551

**Published:** 2014-06-19

**Authors:** Uriel Jeshua Sánchez-Reyes, Santiago Niño-Maldonado, Robert W. Jones

**Affiliations:** 1 División de Estudios de Posgrado e Investigación. Instituto Tecnológico de Cd. Victoria. Boulevard Emilio Portes Gil No.1301, C.P. 87010. Ciudad Victoria, Tamaulipas, México; 2 Facultad de Ingeniería y Ciencias. Universidad Autónoma de Tamaulipas. Centro Universitario Victoria. CP. 87149. Victoria, Tamaulipas, México; 3 Facultad de Ciencias Naturales. Universidad Autónoma de Querétaro. Avenida de las Ciencias, s/n, 76230 Juriquilla, Querétaro, México

**Keywords:** Chrysomelidae, leaf beetles, species richness, abundance, altitude, Northeast Mexico

## Abstract

The Chrysomelidae (Coleoptera) is a highly speciose family that has been poorly studied at the regional level in Mexico. In the present study, we estimated species richness and diversity in oak-pine forest, Tamaulipan thorny scrub and in tropical deciduous forests in Peregrina Canyon within the Altas Cumbres Protected Area of the northeastern state of Tamaulipas, Mexico. Sampling of Chrysomelidae consisted of five sweep net samples (200 net sweeps) within each of three sites during four sample periods: early dry season, late dry season, early wet season, and late wet season. Species were identified and total numbers per species were recorded for each sample. A total of 2,226 specimens were collected belonging to six subfamilies, 81 genera and 157 species of Chrysomelidae from the study area. Galerucinae was the most abundant subfamily with 1,828 specimens, representing 82.1% of total abundance in the study area. Lower abundance was recorded in Cassidinae (8.5%), Eumolpinae (3.6%), Cryptocephalinae (2.2%), Chrysomelinae (2.2%), and finally Criocerinae (1.3%). The highest species richness was also presented in the subfamily Galerucinae with 49% of the total obtained species followed by Cassidinae (20%), Cryptocephalinae (9.7%), Eumolpinae (9.7%), Chrysomelinae (6.5%) and Criocerinae (5.2%). The most common species were *Centralaphthona fulvipennis* Jacoby (412 individuals), *Centralaphthona diversa* (Baly) (248), *Margaridisa* sp.1 (219), *Acallepitrix* sp.1 (134), *Longitarsus* sp.1 (104), *Heterispa vinula* (Erichson) (91), *Epitrix* sp.1 (84) and *Chaetocnema* sp.1 (72). Twenty-two species were doubletons (1.97% of total abundance) and 52 were singletons (2.33%). The estimated overall density value obtained was 0.0037 individuals/m2. The greatest abundance and density of individuals were recorded at the lowest elevation site. However, alpha diversity increased with increasing altitude. Similarity values were less than 50% among the three sites indicating that each site had distinct species assemblages of Chrysomelidae. The highest abundance was obtained during the late dry season, whereas diversity indices were highest during the early wet season. The present work represents the first report of the altitudinal variation in richness, abundance, and diversity of Chrysomelidae in Mexico. These results highlight the importance of conservation of this heterogeneous habitat and establish baseline data for Chrysomelidae richness and diversity for the region.

## Introduction

Chrysomelidae is one of the largest families within the order Coleoptera, with over 35,000 species described worldwide (Jolivet et al. 2009). In Mexico, about 2,174 species are known (Ordóñez-Reséndiz et al. 2014), although the actual number is probably considerably greater. The family is also an economically important group due to their predominantly phytophagous feeding habits (Ding et al. 2007, Meissle et al. 2009). This feeding characteristic and their generally high abundance also make leaf beetles an important component of food webs and a major component of tropical herbivore guilds (Farrell and Erwin 1988, Basset and Samuelson 1996) as well as being an important food item for other organisms (Eben and Barbercheck 1996).

The great species richness of Chrysomelidae and their role as a phytophagous functional group make the Chrysomelidae a potentially useful indicator group for: 1) biodiversity of a region (Farrell and Erwin 1988, Kalaichelvan and Verma 2005, Baselga and Novoa 2007, Aslan and Ayvaz 2009), 2) environmental quality (Linzmeier et al. 2006), and 3) as a taxon for monitoring changes in natural areas (Staines and Staines 2001, Flowers and Hanson 2003). However, the use of this family as such has not been adequately explored. In addition, the general lack of published studies of the species richness and diversity of Chrysomelidae in Mexico (Burgos-Solorio and Anaya-Rosales 2004, Andrews and Gilbert 2005, Niño et al. 2005, Furth 2006, Furth 2009), makes it difficult to compare the particular ecological characteristics and biogeographical distribution patterns of this family with other taxa in the country.

Recent climatic and environmental changes create an ecological imbalance that threatens biodiversity. It is vital that baseline data is available through faunistic inventories along elevational gradients to record and predict how organisms alter distributions and adapt to environmental changes (Maveety et al. 2011). This is especially so for Mexico where altitude is often associated with marked changes in the richness and abundance of species (Peterson et al. 1993), producing rapidly changing distribution patterns along altitudinal gradients (Hodkinson 2005).

The present study was conducted in the Cañon of the Peregrina within Altas Cumbres Protected Area (Vargas et al. 2001) within the northeastern state of Tamaulipas, Mexico. This protected area is located in one of the 15 panbiogeographic nodes in the country. These nodes have unique characteristics which make them centers of higher species richness with high conservation priority (Morrone and Márquez 2008) making the study area an excellent site for analysis of biodiversity and altitudinal distribution of Chrysomelidae in northern Mexico.

The objectives of the present study were: 1) determine the species richness of Chrysomelidae in Peregrina Canyon, Tamaulipas, Mexico; 2) conduct the first site-specific evaluation of diversity for this taxon in northeast Mexico; and 3) analyze the variation of species richness, abundance and diversity of the family along an altitudinal gradient during different seasons within the study area.

## Methods

### Study area

**Figure 1.**
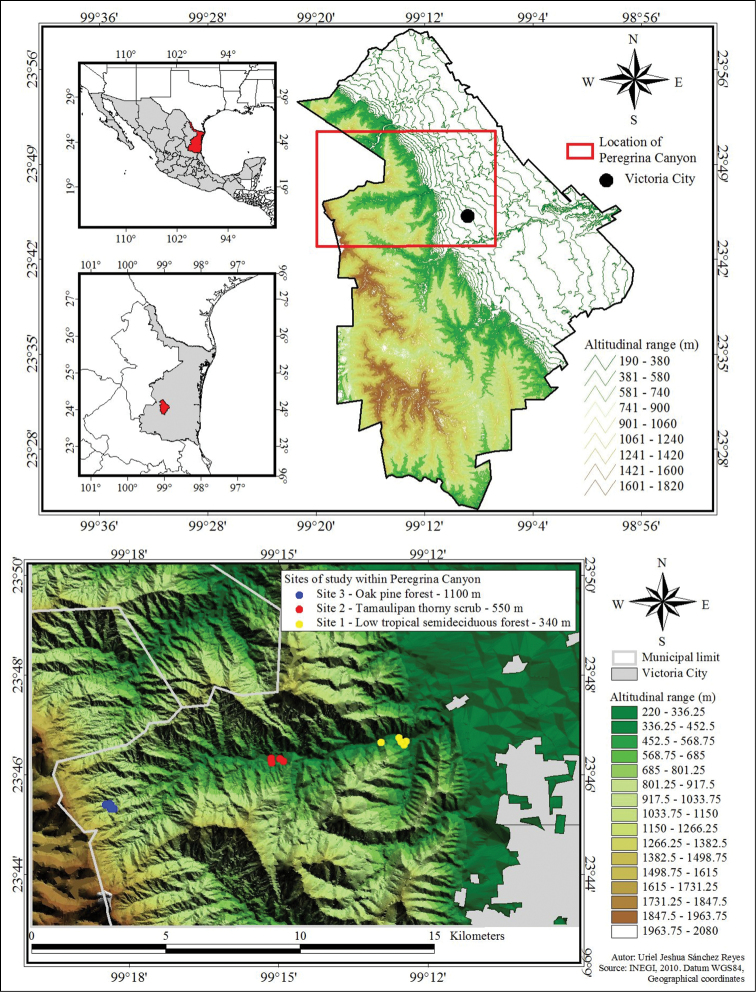
Location of Peregrina Canyon in Tamaulipas, Mexico, and location of sampling sites along study area.

The Peregrina Canyon (Canyon San Felipe or Liberty), is located in the northwest portion of municipality of Victoria, Tamaulipas, along the San Felipe River (Figure 1). The area is located in the Sierra Madre Oriental and is part of Altas Cumbres Protected Area, considered a Special Zone subject to Ecological Conservation established by state decree in 1997 (Vargas et al. 2001). The study area belongs to one of the 15 panbiogeographic nodes present in the country due to the overlap of three biotic provinces: Tamaulipas, Sierra Madre Oriental, and Mexican Gulf (Morrone and Márquez 2008). The altitude within the study area ranges from 340 to 1600 m. The climate of the region is warm and subhumid with summer rains; the mean annual temperature is 18 to 24.3 °C and the mean total annual rainfall is 717.3 mm to 1058.8 mm (Almaguer-Sierra 2005).

### Site location

Three sites were established within which five quadrants of 2500 m^2^ (50×50 m) were delineated in representative vegetation at each site. Site 1 had the lowest elevation at 340 m and consisted of low tropical semideciduous forest (23°45.30'N, 99°18.39'W). Site 2 was located at an intermediate altitude at 550 m where the plant community consisted of Tamaulipan thorny scrub (23°46.32'N, 99°14.96'W). Site 3 was the highest site at 1100 m with the vegetation composed of oak-pine forest (23°46.62'N, 99°12.55'W).

### Collection and processing of specimens

Sampling was conducted using a standard entomological sweep net of 40 cm diameter. Individual samples consisted of 200 sweeps of the shrub and herbaceous vegetation in each quadrant. The contents of the net were emptied into a 2000 cm^3^ plastic bag, adding 60% ethanol and an indelible label with corresponding data. Samples were collected at each site from 10:00 to 14:00 hrs. Five samples (one sample for each quadrant, 200 net sweeps) were taken within each of three sites (one site per day), at four different dates in each of the four seasons of the year (early dry season, EDR, December-February; late dry season, LDS, March-May; early wet season, EWS, June-August; and late wet season, LWS, September-November) between January and December 2009, for a total of 240 samples.

Processing of the samples was performed in the laboratory in the following manner. First, the contents of each plastic bag (sample) were placed in a plastic tray with water, and the more voluminous plant remains (wood fragments, branches, stems, leaves) were removed. A sieve ALSA (0.175 mm) was then used to filter the sample, and the reduced contents placed in a petri dish and observed under a stereoscopic microscope for extraction of all chrysomelid beetles. These were separated and mounted on paper points according to standard entomological technique. All specimens are stored in the collection of the Facultad de Ingeniería y Ciencias at the Universidad Autónoma de Tamaulipas, Ciudad Victoria, Tamaulipas, Mexico.

### Taxonomic determination

The identification of the specimens was performed using the available literature on Chrysomelidae (Wilcox 1965, White 1968, Wilcox 1972, Scherer 1983, White 1993, Flowers 1996, Riley et al. 2002, Staines 2002). Where possible, the material was compared with identified specimens deposited in the collection of Chrysomelidae of the Facultad de Ingeniería y Ciencias, Universidad Autónoma de Tamaulipas. Those specimens that could not be identified to the species level were compared with other unidentified specimens and grouped into morphospecies. The designation of “species” in this study includes both morphospecies and determined species. The classification used in this work corresponds to latest taxonomic categories proposed by Riley et al. (2003), except for the subfamily Bruchinae not included in this study.

### Data analysis

Abundance was calculated using the number of individuals per species collected at each site, season and for the entire study area. Species abundance was divided into five categories: 1) very common (more than 70 individuals); 2) common (11 to 70); 3) rare (10 to three specimens); 4) doubletons (two specimens); and 5) singletons (one specimen only). As a measure of species richness, we used the number of species present throughout the Peregrina Canyon, in each of the three altitudinal strata analyzed, and in each season. To estimate the potential number of species (total, site, and season), the nonparametric estimators Chao 1 and Jackknife 1 were used. These estimators were chosen because: 1) we did not assume a previous abundance distribution model, 2) they are robust when calculating minimum estimate of species richness, 3) their use is recommended as a recurrent measure in analysis of biodiversity, 4) Chao 1 is based on abundance data, or singletons and doubletons, and Jackknife 1 (incidence) is based on uniques, or species found in only one sample (Magurran 2004, Hortal et al. 2006, Gotelli and Colwell 2010), and 5) Jackknife indices tend to be conservative estimators, so the use of both Chao 1 and Jackknife 1 can give an estimated range of species richness (Silva and Coddington 1996). The estimators were calculated with 100 randomizations without replacement using the software EstimateS 8.2 (Colwell 2009) based on the number and abundance of species found per sampling unit (quadrant). Sampling efficiency was also measured by using Clench model, through the coefficient of determination (R^2^) and the slope of the species accumulation curve, which measures the inventory quality. Their calculation was based on the number of samples (quadrants) in the entire study area, site and season; the procedure was performed in the program STATISTICA 8.0 (StatSoft Inc. 2007) based on the method described by Jiménez-Valverde and Hortal (2003). We also calculated overall density, or number of chrysomelid beetles per square meter for future comparisons and was calculated for the entire study area and for each site and season.

After testing for normality of the data, we used the nonparametric Kruskall Wallis and Mann-Whitney tests to analyze the differences in abundance and number of species among the three sites and between different seasons (PAST version 1.94b, Hammer et al. 2001) using as independent variables the total number of specimens and species per sample unit (quadrant).

Alpha diversity for the whole study area and by site and season was calculated using the Simpson diversity index (1/D) and the Shannon diversity index (H ‘) (Magurran 2004), using EstimateS 8.2. Differences of diversity values between sites and seasons were analyzed using PAST version 1.94b (Hammer et al. 2001). SHE analysis of diversity was conducted to decompose the Shannon diversity value in a measure of species richness and evenness, to allow the interpretation of changes in diversity (Magurran 2004). As a beta diversity measure, Bray-Curtis similarity index (Sorensen’s quantitative index; Magurran 2004) was used among the sites and seasons, using EstimateS 8.2; these data were used to build a distance matrix for an agglomerative cluster analysis, using the Ward´s method as amalgamation algorithm calculated using STATISTICA 8.0. A Spearman correlation test was applied between precipitation and temperature data with ecological parameters (abundance and species richness) using STATISTICA 8.0. Precipitation and temperature data were obtained from a local meteorological station localized in Peregrina Canyon.

## Results

### Abundance, richness and diversity of Chrysomelidae in Peregrina Canyon

A total of 2,226 specimens of Chrysomelidae were collected from 240 samples from May 2009 to April 2010, belonging to six subfamilies, 81 genera and 157 species (Table 1). Galerucinae was the most abundant subfamily with 1,828 specimens, representing 82.1% of total abundance in the study area. Lower abundance was recorded in Cassidinae (8.5%), Eumolpinae (3.6%), Cryptocephalinae (2.2%), Chrysomelinae (2.2%), and finally Criocerinae (1.3%). The highest species richness was also presented in the subfamily Galerucinae with 49% of the total obtained species followed by Cassidinae (20%), Cryptocephalinae (9.7%), Eumolpinae (9.7%), Chrysomelinae (6.5%) and Criocerinae (5.2%).

**Table 1.** Taxonomic list and abundance of Chrysomelidae by season and site in Peregrina Canyon, Tamaulipas, Mexico. N = Total abundance; 1 = Low tropical semideciduous forest, 340 m; 2 = Tamaulipan thorny scrub, 550 m; 3 = Oak-pine forest, 1100 m.

	Dry Season						Wet Season						
	Early (Dec-Feb)			Late (Mar-May)			Early (Jun-Aug)			Late (Sep-Nov)			N
	1	2	3	1	2	3	1	2	3	1	2	3	
**CASSIDINAE Gyllenhal, 1813**													
**Tribu Chalepini Weise, 1910**													
*Anisostena pilatei* (Baly, 1864)					1	1		1					**3**
*Brachycoryna pumila* Guérin-Méneville, 1844	1	3			4	1		5					**14**
*Chalepusbellulus* (Chapuis, 1877)													**5**
*Chalepus digressus* Baly, 1885					1								**1**
*Euprionota aterrima* Guérin-Méneville, 1844									1				**1**
*Glyphuroplata* sp. 1					1								**1**
*Heterispavinula* (Erichson, 1847)	1	6		1	20	8	1	29	1	10	12	2	**91**
*Octotoma championi* Baly, 1885				2									**2**
*Octotoma intermedia* Staines, 1989					1								**1**
*Sumitrosis inaequalis* (Weber, 1801)		2			2			1					**5**
*Sumitrosis pallescens* (Baly, 1885)						1							**1**
*Sumitrosisrosea* (Weber, 1801)				1	1		1		3	2			**8**
*Sumitrosis* sp. 1								1		1			**2**
*Sumitrosis* sp. 2												1	**1**
*Xenochalepus (Neochalepus) chapuisi* (Baly, 1885)				1									**1**
*Xenochalepus (Xenochalepus) omogerus* (Crotch, 1873)								1	1				**2**
**Tribu Cassidini Gyllenhal, 1813**													
*Charidotella bifossulata* (Boheman, 1855)						1		3	1				**5**
*Charidotella (Chaerocassis) emarginata* (Boheman, 1855)									1				**1**
*Charidotella sexpunctata* (Fabricius, 1781)							3	1					**4**
*Charidotella tuberculata* (Fabricius, 1775)				2									**2**
*Charidotis auroguttata* Boheman, 1855					1								**1**
**Tribu Cassidini Gyllenhal, 1813**													
*Charidotellatuberculata* (Fabricius, 1775)				2									**2**
*Charidotis auroguttata* Boheman, 1855					1								**1**
*Coptocycla (Psalidonota) texana* (Schaeffer, 1933)								2					**2**
*Microctenochirapunicea* (Boheman, 1855)				1		2			2				**5**
*Microctenochira varicornis* (Spaeth, 1926)						1							**1**
*Microctenochiravivida* (Boheman, 1855)				1		1							**2**
*Helocassis crucipennis* (Boheman, 1855)						9		3	1		1		**14**
*Helocassis testudinaria* (Boheman, 1855)				1			1	1	2				**5**
**Tribu Mesomphaliini Hope, 1840**													
*Chelymorpha pubescens* Boheman, 1854				1									**1**
*Hilarocassis exclamationis* (Linnaeus, 1767)								1					**1**
*Ogdoecosta juvenca* (Boheman, 1854)								3	1	2			**6**
**Tribu Ischyrosonychini Chapuis, 1875**													
*Physonota alutacea* Boheman, 1854								1					**1**
**CHRYSOMELINAE Latreille, 1802**													
**Tribu Chrysomelini Latreille, 1802**													
**Subtribu Doryphorina Motschulsky, 1860**													
*Calligrapha fulvipes* Stål, 1859								1					**1**
*Calligrapha* sp. 1								1					**1**
*Calligrapha* sp. 2				2									**2**
*Calligrapha suffriani* Jacoby, 1882								1					**1**
*Labidomera suturella* Chevrolat, 1844							1	3	3				**7**
*Zygogramma piceicollis* (Stål, 1859)	1												**1**
**Subtribu Chrysomelina Latreille, 1802**													
*Chrysomela texana* (Schaeffer, 1919)				2			3						**5**
*Phaedon cyanescens* Stål, 1860							1						**1**
*Plagiodera semivittata* Stål, 1860	3			3	1				1	2	2		**12**
*Plagiodera thymaloides* Stål, 1860	1	2			5	1				9			**18**
**CRIOCERINAE Latreille, 1807**													
**Tribu Lemini Heinze, 1962**													
*Lema balteata* LeConte, 1884								1					**1**
*Lema* sp. 1						2					1	1	**4**
*Neolema quadriguttata* White, 1993				2		1		1					**4**
*Neolema* sp. 1					1								**1**
*Neolema* sp. 2					1	1							**2**
*Neolema* sp. 3				2		1						1	**4**
*Oulema* sp. 1				4		3			2			3	**12**
*Oulema* sp. 2										1			**1**
**CRYPTOCEPHALINAE Gyllenhal, 1813**													
**Tribu Cryptocephalini Gyllenhal, 1813**													
**Subtribu Cryptocephalina Gyllenhal, 1813**													
*Cryptocephalus duryi* Schaeffer, 1906								1				2	**3**
*Cryptocephalus* sp. 1	1												**1**
*Cryptocephalusumbonatus* Schaeffer, 1906								2		1	1		**4**
*Diachus auratus* (Fabricius, 1801)			5			8			2				**15**
**Subtribu Pachybrachina Chapuis, 1874**													
*Pachybrachis* sp. 1	1									1			**2**
*Pachybrachis* sp. 2		1			1								**2**
*Pachybrachis* sp. 3		1			1	1		1					**4**
*Pachybrachis* sp. 4		2									1		**3**
*Pachybrachis* sp. 5										2			**2**
*Pachybrachis* sp. 6												1	**1**
*Pachybrachis* sp. 7					1						1		**2**
**Tribu Clytrini Lacordaire, 1848**													
**Subtribu Clytrina Lacordaire, 1848**													
*Anomoea rufifrons* Chevrolat, 1837								1	4				**5**
**Subtribu Megalostomina Chapuis, 1874**													
*Coscinoptera scapularis scapularis* (Lacordaire, 1848)								1					**1**
*Coscinopteravictoriana* L. Medvedev, 2012								1					**1**
**Subtribu Babiina Chapuis, 1874**													
*Babia tetraspilota* LeConte, 1858							2						**2**
**Tribu Chlamisini Gressitt, 1946**													
*Chlamisus texanus* (Schaeffer, 1906)				3									**3**
*Neochlamisus* sp. 1									1				**1**
**EUMOLPINAE Hope, 1840**													
**Tribu Eumolpini Hope, 1840**													
*Brachypnoea* sp. 1				9		9							**18**
*Brachypnoea* sp. 2				9	2	2			1				**14**
*Chalcophana cincta* Harold, 1874									1			4	**5**
*Colaspis melancholica* Jacoby, 1881								1					**1**
*Colaspis* sp. 1											1		**1**
*Colaspistownsendi* Bowditch, 1921										4			**4**
*Tymnes* sp. 1				1				1		1			**3**
*Zenocolaspis inconstans* Bechyné, 1997										3			**3**
**Tribu Adoxini Baly, 1863**													
*Fidia albovittata* Lefèvre, 1877						1							**1**
*Xanthonia* sp. 1		1	5	1		3					1		**11**
*Xanthonia* sp. 2		3											**3**
*Xanthonia* sp. 3					1	4							**5**
*Xanthonia* sp. 4				1		7							**8**
*Xanthonia* sp. 5	1	1											**2**
**Tribu Typophorini Chapuis, 1874**													
*Typophorusnigritus* (Fabricius, 1801)										1			**1**
**GALERUCINAE Latreille, 1802**													
**Tribu Alticini Newman, 1835**													
*Acallepitrix* sp. 1	2			35	8	82	1	2	3			1	**134**
*Acallepitrix* sp. 2	1	1		15	1	3	2		3				**26**
*Acallepitrix* sp. 3	3			3	1	2		3	2	5	4	2	**25**
*Acallepitrix* sp. 4						4			4				**8**
*Acallepitrix* sp. 5									1				**1**
*Acrocyum dorsalis* Jacoby, 1885					1								**1**
*Alagoasa bipunctata* (Chevrolat, 1834)				1				1			1		**3**
*Alagoasa decemguttatus* (Fabricius, 1801)				1	2	1		4	1			1	**10**
*Alagoasa* sp. 1					1	4							**5**
*Asphaera abdominalis* (Chevrolat, 1834)								8	2			2	**12**
*Asphaera* sp. 1								13	1				**14**
*Asphaera* sp. 2								1					**1**
*Asphaera* sp. 3								1					**1**
*Asphaera* sp. 4				1									**1**
*Blepharida rhois* (Forster, 1771)					1								**1**
*Centralaphthona diversa* (Baly, 1877)	6	80	6	1	52	30		14		33	25	1	**248**
*Centralaphthona fulvipennis* (Jacoby, 1885)	309	1		42			3			56		1	**412**
*Chaetocnema* sp. 1	27	12	12	9	9			2			1		**72**
*Chaetocnema* sp. 2			1	1	1	5							**8**
*Chaetocnema* sp. 3	5	5	2		1	7		3	2				**25**
*Derocrepis* sp. 1				5									**5**
*Derocrepis* sp. 2						2							**2**
*Disonycha antennata* Jacoby, 1884								1					**1**
*Disonycha glabrata* (Fabricius, 1781)				5	1			1				1	**8**
**Tribu Alticini cont**													
*Disonycha stenosticha* Schaeffer, 1931								1		1			**2**
*Epitrix* sp. 1	20	7		15	9		7		1	20	2	3	**84**
*Epitrix fasciata* Blatchley, 1918	2				2				1			2	**7**
*Epitrix* sp. 3			2			2					1	7	**12**
*Glenidion* sp.1		1							1				**2**
*Heikertingerella* sp. 1						1				1			**2**
*Heikertingerella* sp. 2			4										**4**
*Heikertingerella variabilis* (Jacoby, 1885)	4			1		1				1			**7**
*Longitarsus* sp. 1	8	36		25	14	8	2	9	2				**104**
*Longitarsus* sp. 2								1					**1**
*Longitarsus* sp. 3	1	1											**2**
*Longitarsus* sp. 4				1									**1**
*Longitarsus* sp. 5	1	2		8	8			2					**21**
*Lupraea* sp. 1			1							13		18	**32**
*Lupraea* sp. 2										8			**8**
*Lupraea* sp. 3										16	1	7	**24**
*Lupraea* sp. 4										26	6	1	**33**
*Lysathia* sp. 1				1									**1**
*Margaridisa* sp. 1	52	2	3	61	27		37	5		29	3		**219**
*Margaridisa* sp. 2	1						1			1		3	**6**
*Monomacra* sp. 1	5	4		2	5		3	2		5	3	4	**33**
*Monomacra* sp. 2						6							**6**
*Monomacra* sp. 3				1									**1**
*Omophoita cyanipennis octomaculata* (Crotch, 1873)								4			2		**6**
*Orthaltica* sp. 1						5							**5**
*Orthaltica* sp. 2			1										**1**
*Parchicola* sp. 1	2									1			**3**
*Phyllotreta* sp. 1	3	1	2	1	1								**8**
*Plectrotetra* sp. 1					3	13		2					**18**
*Scelidopsis rufofemorata* Jacoby, 1888				1									**1**
**Tribu Alticini cont**													
*Sphaeronychusfulvus* (Baly, 1879)			1			1			2	1			**5**
*Strabala* sp. 1					1								**1**
*Syphrea* sp. 1		2		10	8		1	1					**22**
*Syphrea* sp. 2				1	2	6		1	1	1	1		**13**
*Systena contigua* Jacoby, 1884					1	19		7					**27**
*Systena* sp. 1				1									**1**
*Walterianella signata* (Jacoby, 1886)					2			1					**3**
**Tribu Galerucini Latreille, 1802**													
**Grupo Coelomerites Chapuis, 1875**													
*Coraiasubcyanescens* (Schaeffer, 1906)				1				1	2	1			**5**
*Derospidea cyaneomaculata* (Jacoby, 1886)											1		**1**
*Trirhabda* sp. 1						1							**1**
**Grupo Schematizites Chapuis, 1875**													
*Ophraearugosa* Jacoby, 1886						1			1				**2**
**Tribu Luperini Chapuis, 1875**													
**Subtribu Diabroticina Chapuis, 1875**													
**Grupo Diabroticites Chapuis, 1875**													
*Acalymma vittatum* (Fabricius, 1775)						1		1					**2**
*Diabroticabalteata* LeConte, 1865				1				1	1				**3**
*Diabrotica porracea* Harold, 1875						1							**1**
*Diabroticaunderwoodi* Bowditch, 1911				1		7		2					**10**
*Gynandrobrotica lepida* (Say, 1835)	5		1	13	9	5		1	4	1		1	**40**
**Grupo Cerotomites Chapuis, 1875**													
*Cerotoma atrofasciata* Jacoby, 1879				1									**1**
*Cerotoma ruficornis* (Olivier, 1791)				1									**1**
*Cyclotrypemafurcata* (Olivier, 1808)								3			4		**7**
*Neobrotica sexmaculata* Jacoby, 1887								1					**1**
*Neobrotica tampicensis* Blake, 1966									2				**2**
**Subtribu Luperina Chapuis, 1875**													
**Grupo Monoleptites Chapuis, 1875**													
*Calomicrus* sp. 1		1											**1**

Eight species were categorized as “very common” in the Peregrina Canyon, each with greater than 70 specimens and accounted for 61.22% of the total abundance. These very common species were *Centralaphthona fulvipennis* Jacoby (412 individuals), *Centralaphthona diversa* (Baly) (248), *Margaridisa* sp.1 (219), *Acallepitrix* sp.1 (134), *Longitarsus* sp.1 (104), *Heterispa vinula* (Erichson) (91), *Epitrix* sp.1 (84) and *Chaetocnema* sp.1 (72). Twenty-five species were considered common, constituting 22.66% of the total number of chrysomelids. Fifty species were considered rare (263 specimens) by occupying 11.8% of the total abundance. Twenty-two species were doubletons (1.97% of total abundance) and 52 were singletons (2.33%). The estimated density value obtained was 0.0037 individuals/m^2^ (Table 2).

**Table 2.** Richness, abundance and diversity parameters of Chrysomelidae in the Peregrina Canyon, Tamaulipas, Mexico. S obs = Observed richness; N = Abundance; Dst = Density; S est = Estimated richness; R^2^ = Clench model determination coefficient; 1/D = Simpson diversity index; H´= Shannon diversity index.

Ecological Parameter	Site			Season				Total
	Low tropical semideciduous forest	Tamaulipan thorny scrub	Oak-pine forest	Dry		Wet		
				Early (Dec-Feb)	Late (Mar-May)	Early (Jun-Jul)	Late (Aug-Nov)	
S obs ^†^	85a	96ab	84b	43c	96a	84b	56bc	157
N ^†^	1123a	641b	464c	696c	822a	304b	406bc	2228
Dst	0.0056	0.0032	0.0023	0.0046	0.0054	0.002	0.0027	0.0037
**S est**								
Chao 1	119.13	196.04	140.89	49.05	132	134.7	84.41	218.45
Jackknife 1	126.48	150.31	123.5	55.78	137.3	130.22	84.52	216.75
**Clench**								
R^2^	0.997	0.998	0.999	0.998	0.998	0.999	0.999	0.997
S est	128.49	171.80	133.91	57.70	143.46	175.59	94.85	212.41
Slope	0.368	0.532	0.395	0.187	0.539	0.729	0.385	0.178
**Diversity** ^‡^								
1/D	5.95a	10.12b	19.26c	4.28d	17.31a	24.66b	13.92c	14.6
H’	2.73a	3.24b	3.67c	2.17d	3.49a	3.78b	3.06c	3.54

^†^ Values with different letters within rows are significantly different using Kruskal-Wallis and Mann-Whitney Tests: abundance between sites, K=15.92, DF=2, *p*=0.0003; richness between sites, K=8.17, DF=2, *p*=0.0157; abundance between seasons, K=42.42, DF=3, *p*=0.000; richness between seasons, K=50.15, DF=3, *p*=0.000.

^‡^ Diversity values with different letters within rows are significantly different at *p*<0.05, using permutation and bootstrap tests in PAST program.

**Figure 2.**
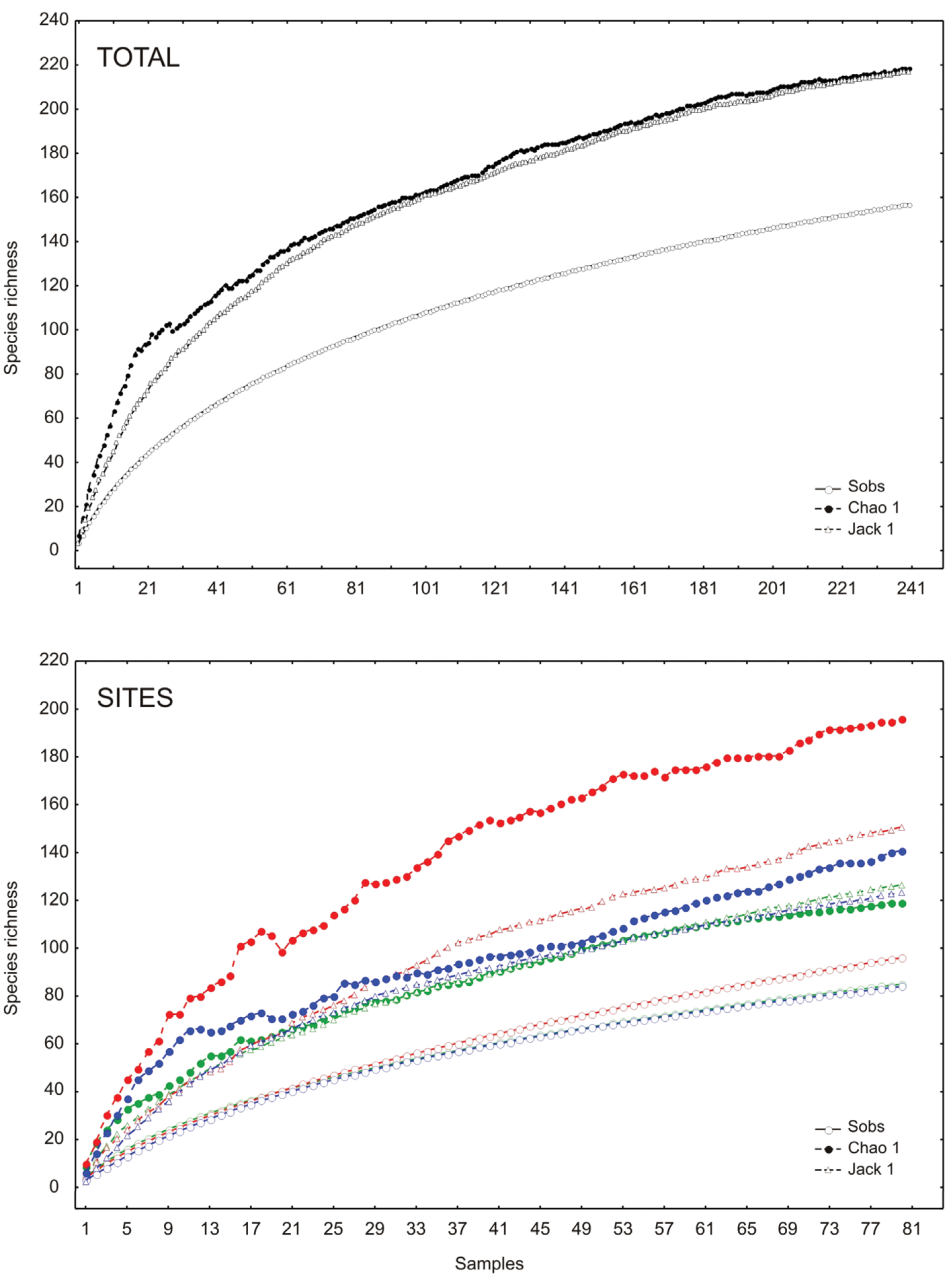
Species accumulation curves by altitudinal site in the Peregrina Canyon, Tamaulipas, Mexico. Upper graphic: accumulation curves for all study area. Lower graphic: site **1** (green color), site **2** (red color) and site **3** (blue color).

**Figure 3.**
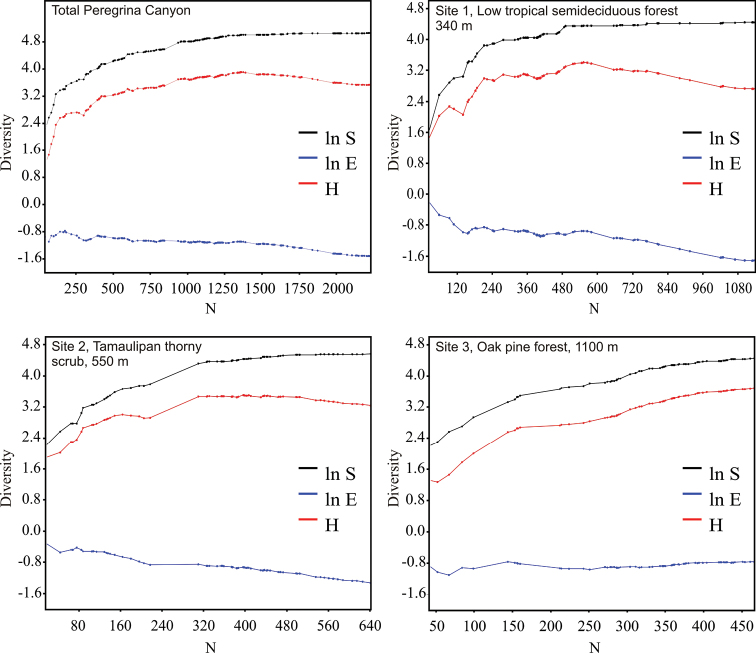
SHE analysis of diversity for the Peregrina Canyon and for each one of altitudinal sites. ln **S** natural logarithm of species richness; ln **E** natural logarithm of evenness; **H** diversity (Shannon index).

The richness estimators indicated that the total number of chrysomelid species in the study area was between 216 and 218 species (Table 2, Figure 2) suggesting that the observed total of 157 species represented 71.86 to 72.43% of the actual richness. The data showed a good fit to the Clench model (R^2^ = 0.99), with a registered proportion of species of 73.91% and a slope close to 0.1. Total diversity values of Chrysomelidae in Peregrina Canyon were 14.58 for the Simpson index and 3.53 for the Shannon index (Table 2). The SHE analysis shows that changes in Shannon diversity value are attributed to increase and stability of species richness curve (Figure 3).

### Altitudinal variation of Chrysomelidae

The abundance of chrysomelid beetles was significantly different among the three sites (Table 2). The greatest abundance and density (individuals per square meter) were recorded at the lowest elevation site, and decreased with increasing altitude (Table 2). The middle altitude site (Site 2) had the greatest number of species (Table 2). The number of species significantly differed only between the lowest and the highest altitudinal strata (Site 1 and Site 3; Table 2). In the low altitude site, 85 species were recorded which represented between 67.2 to 71.35% of the estimated richness (minimum and maximum) with the models used. In the second site, the number increased to 96 species (48.96 to 63.86% of the estimate) and at the highest site, 84 species were recorded (59.62 to 68.01% of the estimate) (Figure 2). A determination coefficient greater than 0.99 was obtained for all sites, indicating a good fit of the Clench model to the data obtained at each site, but the slope calculated was greater than 0.1 in all sites (Table 2).

**Figure 4.**
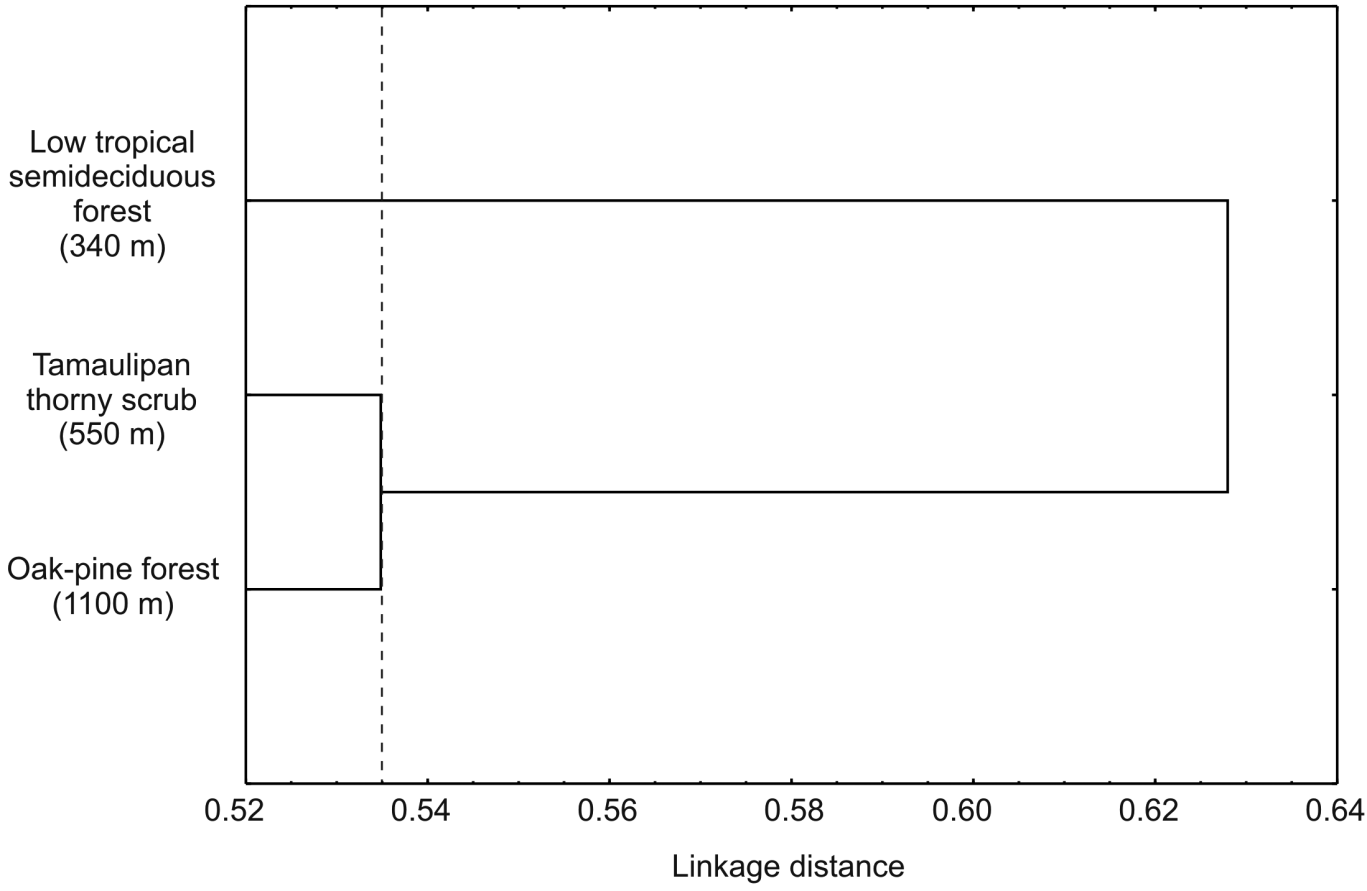
Cluster analysis from sites in the Peregrina Canyon, Tamaulipas, Mexico.

Alpha diversity at the three sites differed significantly (*p* < 0.05) with indices increasing progressively with increasing altitude (Table 2). Lower diversity values in both sites 1 and 2, were a result of a reduction in eveness and a more or less stable number of species with the increase of samples. In site 3, diversity increases as eveness remained constant and the number of species increased with sample numbers (Figure 3). Of the 157 species recorded in the Peregrina Canyon, 34 were distributed along the entire altitudinal gradient, 40 were recorded only in two sites, and 83 were unique to one of the three sites. Of these, 29 were exclusively from Site 1, 34 for Site 2, and 20 for Site 3 (Table 1). Similarity values were in all cases less than 50%; according to the cluster analysis, each of the three sites was an independent group, containing distinct species assemblages of Chrysomelidae (Figure 4).

### Seasonal variation of Chrysomelidae

**Figure 5.**
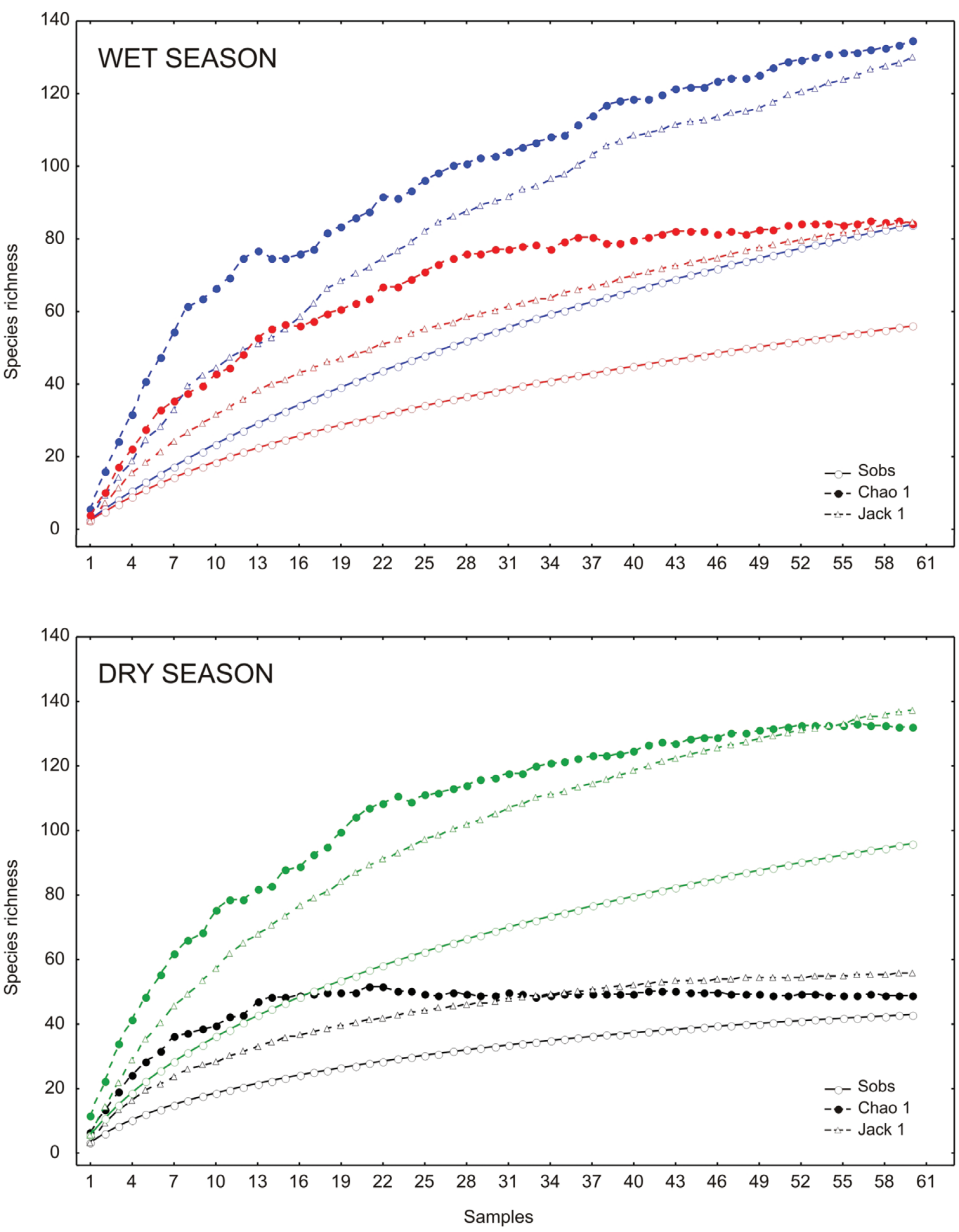
Species accumulation curves by season in the Peregrina Canyon, Tamaulipas, Mexico. Upper graphic: Early wet season (blue color) and late wet season (red color). Lower graphic: Early dry season (black color) and late dry season (green color).

**Figure 6.**
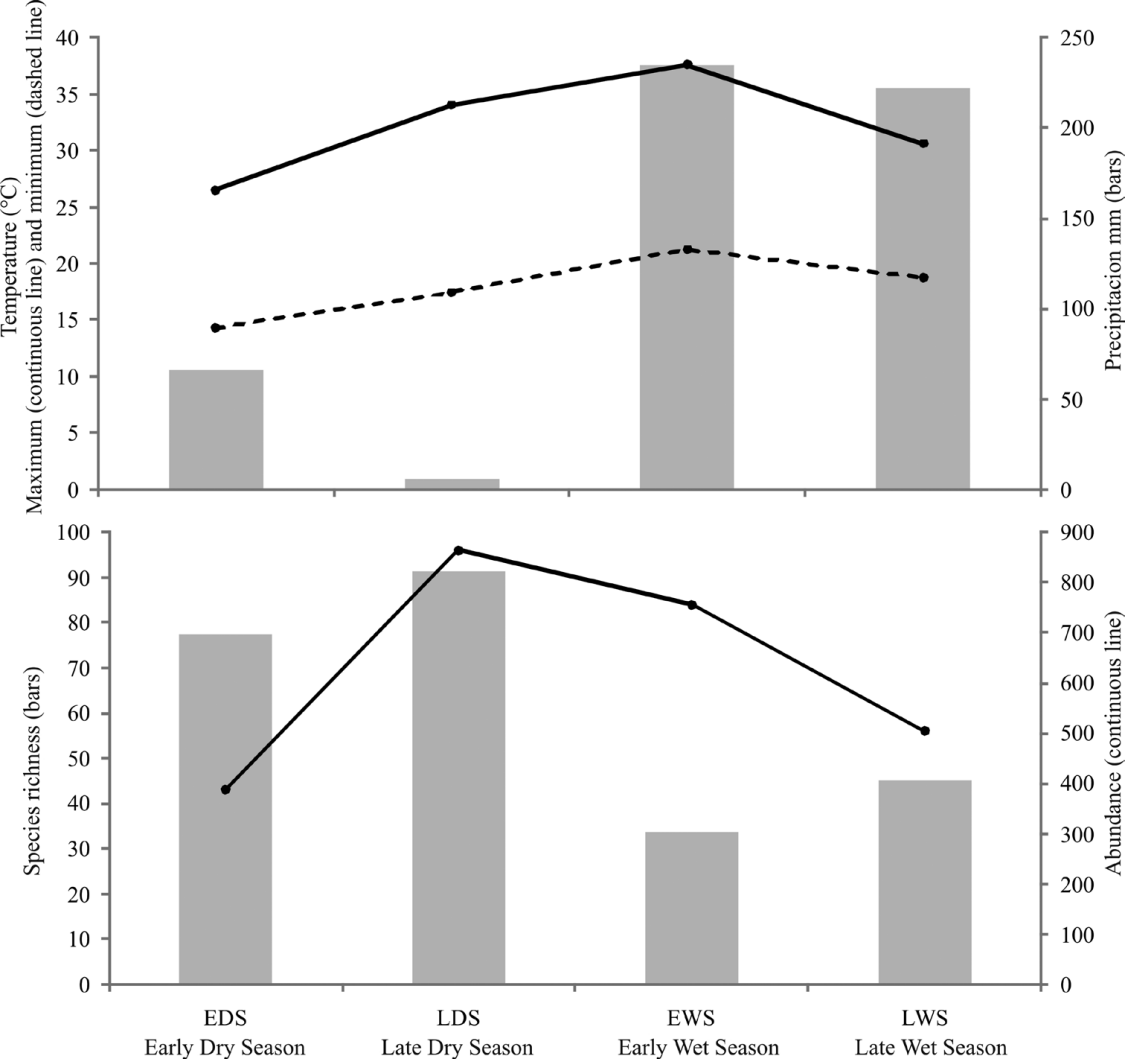
Variation of Chrysomelidae with precipitation and temperature during 2009 in Peregrina Canyon.

General abundance of Chrysomelidae was greater in the dry season than the wet season. Late wet season was not significantly different from early dry and wet seasons; the rest of comparisons between seasons were significantly different. The highest abundance was obtained during the late dry season, with 822 individuals. Fewer individuals were found during the early dry season (696 specimens), and late and early wet seasons, 406 and 302, respectively. Density for seasons followed the same pattern as the abundance, being late dry season the period with higher densities of chrysomelid beetles (Table 2). The number of species collected per season declined as the year progresses. During late dry season, 96 species were recorded, representing between 69.91 and 72.72% of the estimated richness for that season; 84 species were found in early wet season (62.36 to 64.5% of estimated richness), while the number decreases to 56 species in late wet season (66.25 to 66.34%) and 43 species in early dry season (77.08 to 87.66%) (Table 2; Figure 5). Determination coefficients based on the Clench model was higher than 0.99 for all seasons, while the slope values were above 0.1 (Table 2). Higher temperatures and precipitation were found within both wet seasons (Figure 6). High correlation values were present between temperature and richness, and between precipitation and abundance. Abundance was negatively correlated with precipitation, while species richness was positively correlated with maximum temperature. Other comparisons were not significant (Table 3).

**Table 3.** Spearman rank order correlations of abundance and species richness of Chrysomelidae with precipitation and temperature in Peregrina Canyon; marked (*) correlations are significant at *p* < 0.05.

	Abundance	Richness
**Precipitation**	-0.769*	0.112
**Max °C**	0.321	0.842*
**Min °C**	-0.066	0.587

**Figure 7.**
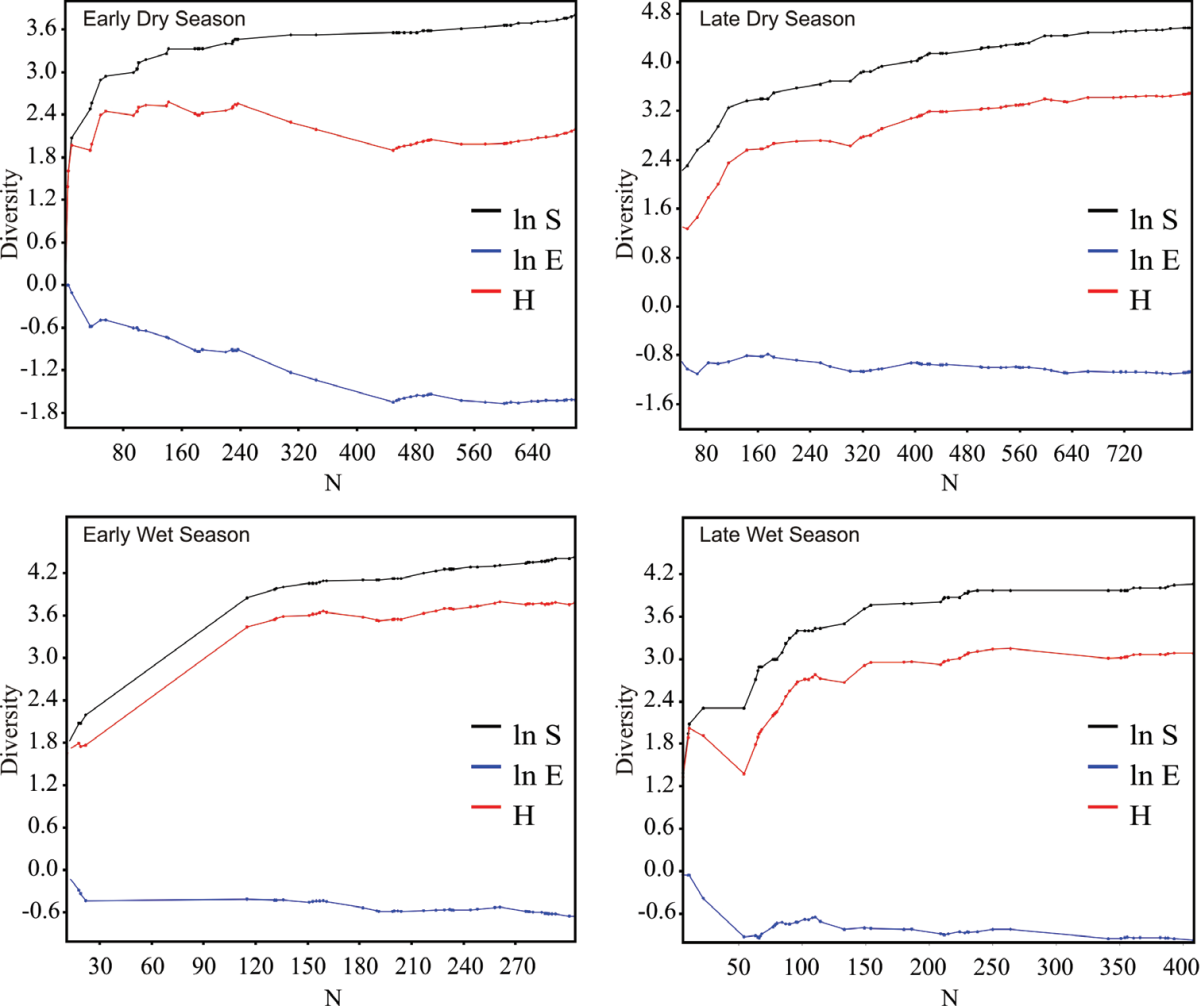
SHE analysis of diversity for each season in the Peregrina Canyon. ln **S** natural logarithm of species richness; ln **E** natural logarithm of evenness; **H** diversity (Shannon index).

**Figure 8.**
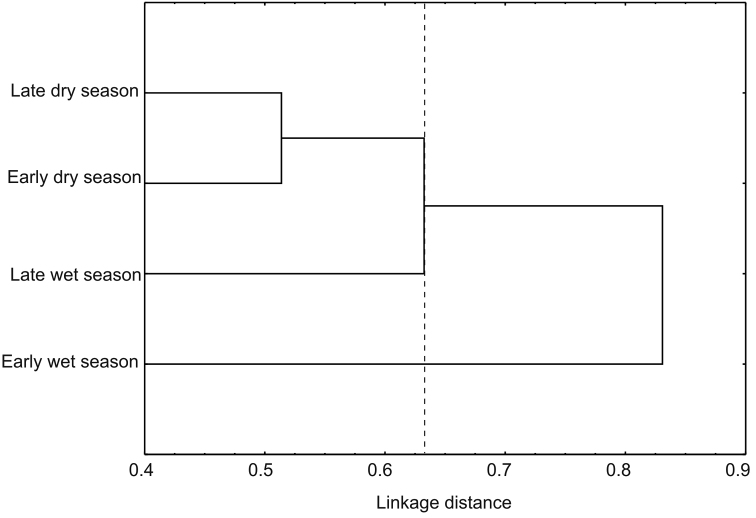
Cluster analysis from seasons in the Peregrina Canyon, Tamaulipas, Mexico.

In contrast to abundance, the Shannon and Simpson indices indicated the highest diversity during the early wet season. Lower values ​​of diversity occurred in late dry season, followed by late wet and early dry seasons. Based on diversity indices, all seasons were statistically different (*p* < 0.05) (Table 2). Reduction in diversity value at early dry season was originated by the drop in evenness with the increase of samples. The rest of year, evenness values, remained constant with the increase of samples in each season (Figure 7). Of the total species recorded in the annual period, only 13 were present throughout the year, and 23 were registered in three seasons, 37 in only two, and 84 were unique to a single season. From these exclusive species, 38 were recorded in late dry season, 26 in early wet season, 12 in late wet season, and only eight in early dry season (Table 1). Bray Curtis index established the greatest similarity between early and late dry seasons (45.1%), and in descending order were presented late wet and early dry seasons (38.8%), late dry and late wet seasons (37.9%), late dry and early wet seasons (36.3%), early and late wet seasons (35.6%) and early wet and early dry seasons (25.7%). The cluster analysis showed the formation of three groups according to the composition of species in each season: the first group consists of the species present in early and late dry seasons, the second group corresponds to late wet season species, and the last group was the early wet season species (Figure 8).

## Discussion

### Species richness in Peregrina Canyon

There are few studies of the richness and diversity of Chrysomelidae in Mexico with which the present study can be compared. Deloya and Ordóñez (2008) reported 136 species in fragments of cloud forest, in the state of Veracruz, while Niño et al. (2005) presented a checklist of 128 species for Biosphere Reserve El Cielo in the state of Tamaulipas, which also included cloud forest vegetation as well as tropical deciduous habitats. Considering that both authors used similar methods to that applied in this research, the 157 species found in this study is noteworthy for its greater richness. This may be a result of the three distinct habitats sampled despite the greater aridness of two of these (thorn scrub and oak forest) when compared to the habitats sampled by Deloya and Ordóñez (2008) and Niño et al. (2005). There are still other studies in Mexico where the species richness or diversity has been analyzed; however, temporal and spatial scale were greater, principally in those studies that were checklists in complete states (Andrews and Gilbert 2005) and natural areas of greater extension (Ordóñez-Reséndiz and López-Pérez 2009, Ordóñez-Reséndiz et al. 2011), or also for all the country and for only one subfamily or tribe, such Chrysomelinae (Burgos-Solorio and Anaya-Rosales 2004), Alticini (Furth 2006, Furth 2009), Cassidinae (Martínez-Sánchez et al. 2010) and Clytrini (Medvedev et al. 2012). Also these studies have been made with very different methodologies and different approaches than in this study which precludes a direct comparison with the results presented here.

Using the latest higher classification scheme of Riley et al. (2003), six subfamilies are formally reported in this study (Table 1). Galerucinae had the greatest number of species which is in accordance with subfamily species totals worldwide (Riley et al. 2002) and in other studies with Chrysomelidae in different parts of the world, including Mexico (Kalaichelvan and Verma 2005, Niño et al. 2005). The 157 species present in this work represent the 7.22% of the 2,174 reported species for México (Ordóñez-Reséndiz et al. 2014), which is a notable percentage considering the small area sampled in this study.

According to both non-parametrics estimators used, our data represent a sampling efficiency superior to 70%. Similarly, Clench model indicated a percentage superior to 70% and a slope close to 0.1, indicating both data accuracy and the reliability of the study (Jimenez-Valverde and Hortal 2003). However, richness estimators indicates that the number of species is greater, thus a complementary method of sampling, such as canopy fogging (Basset and Samuelson 1996, Novotny et al. 1999, Basset 2001, Charles and Basset 2005, Vig and Markó 2005), malaise traps (Flowers and Hanson 2003), or further sweep net samples (Sackmann 2006, Pedraza et al. 2010), would increase the number of species found, as well as reducing the number of singletons and doubletons.

Alpha diversity of Chrysomelidae in Peregrina Canyon was high, and represents one of the first known studies of site specific data for Mexico. Margalef (1972) notes that Shannon index values ​​are typically between 1.5 and 3.5, and rarely exceed a value of 4. Based on this scale, it can be established that Chrysomelidae diversity in the study area is high (Shannon = 3.53). These results can be explained in part by the geographic location of the study area; Peregrina Canyon is located in one of the 15 panbiogeographic nodes of Mexico, within the Sierra Madre Oriental. According to Morrone and Márquez (2008), these areas are centers for high biological diversity, representing the confluence of different biotic provinces. In this case, Peregrina Canyon is in the union of Tamaulipan, Sierra Madre Oriental and Mexican Gulf biotic provinces, thus presenting influences of both temperate and tropical faunas, which harbors high numbers of species in the area and the typical pattern for tropical faunas, with a high percentage of singletons and doubletons (Silva and Coddington 1996, Furth et al. 2003). This confluence of the biotic provinces is evidenced by the biogeographic distribution of the collected species. Considering the distribution for the identified species and the range of the genera for morphospecies, a total of 71 species (both morphospecies and identified species) in this study, (45%) were principally of Neotropical distribution (some ranging up to southern states of USA, but with their major distribution through Mexico and south into Central America and South America). This distribution is shared by many biotic groups of the Mexican Gulf region (Morrone 2005). Another 24 species (15%) had typical Nearctic distribution (Mexico north into the United States and Canada) which in Mexico includes the Tamaulipan province that extends north into Texas. Finally, 56 species (36%) have distributions throughout the American continent (including North, Central and South America), whereas 6 species (4%) were restricted to Mexico and possibly restricted to or have originated within the Sierra Madre Oriental province. Further study of the biogeographical distributions of the Chrysomelidae of Mexico is sorely needed.

### Altitudinal and seasonal variation of Chrysomelidae

We present the first record of the altitudinal variation in richness, abundance, and diversity of Chrysomelidae in Mexico. In our study, greater species richness was found in the intermediate altitudes, which is a similar result to that found by Fernandez et al. (2010) with Staphylinidae, where the number of species increased from first to second altitudinal strata, and then decreased slightly in the highest part of the gradient. Greatest species richness in intermediate altitudes has also been documented in Alticini (Chrysomelidae) (Furth 2009), as well as dung beetles (Celi et al. 2004) and in some species of Scarabaeinae (Escobar et al. 2005). Intermediate areas represent an area of overlap in species distributions, which could explain the higher species richness in this site. Richness estimators in each site indicated inventory completeness of less than 70%, with higher slopes values for Clench model, which indicated a relatively incomplete inventory for each site (Jimenez-Valverde and Hortal 2003). This is due to the high number of doubletons and singletons for each site which influenced the estimators used.

Decreasing abundance with increasing altitude has been observed in several studies with other insects, such as necrophilic entomofauna (Sanchez-Ramos et al. 1993) and ground beetles (Semida et al. 2001). In contrast to abundance, in this study there was a progressive and significant increase in diversity with altitude, which was directly related to the altitudinal abundance recorded in each site. In the first site, the abundance was very high, with some species concentrated in large numbers (e.g., *Centralaphthona fulvipennis*), thus reducing diversity. By contrast, in the higher elevation site, the Chrysomelidae community is represented by a more equitative number of individuals, decreasing dominance and thus increasing values ​​of both diversity indices. This is confirmed by the SHE analysis, where the evenness remain constant and the species richness increases progressively with the increasing samples, being this pattern very different than the other two sites. However, in a similar study with a different phytophagous group, Apionidae (Curculionoidea), Jones et al. (2012) found the opposite pattern with increasing abundance with altitude but decreasing diversity, in the El Cielo Biosfera Reserve, in Tamaulipas, Mexico. Also, Flowers and Hanson (2003) found that both species richness and diversity of Alticini (Chrysomelidae) had lower values on higher altitudes in Costa Rica. However, the pattern found in our study can be explained by the vegetation composition and the plant density in the sampled area, which was higher in the semideciduous tropical forest site, with a greater number of herbaceous plants in the understory. This characteristic is clearly an important factor for Chrysomelidae, because species are almost exclusively phytophagous, with species often highly specialized and thus directly influenced by the composition of the vegetation and the presence and abundance of their host plants (Ribeiro et al. 1994, Řehounek 2002, Aslan and Ayvaz 2009, Flinte et al. 2011, Linzmeier and Ribeiro-Costa 2013).

Novotny et al. (2006) present evidence that the single most important factor contributing to high species diversity of insects in the tropics is the diversity of plants. This would seem to be simplest explanation for the differences in richness, diversity and abundance with altitude of leaf beetles in the present study. Although the abiotic factors that change with altitude, such as precipitation, temperature, air currents, and solar radiation (Lawton et al. 1987, Barry 2008) can certainly affect some aspects of the biology of Chrysomelidae, the single most important factor in the abundance and diversity of these beetles is clearly the presence of their host plants (Ribeiro et al. 1994, Aslan and Ayvaz 2009, Şen and Gök 2009, Flinte et al. 2011). Each site in the present study has markedly different plant communities with different species compositions, densities and vegetation structure. This was reflected in equally different communities and abundances of leaf beetles among the three sites, with less than 50% similarity in species among sites. Adding to the vegetation differences were anthropogenic activities (grazing, logging) observed around some of the quadrants where sampling was carried out. Moderate disturbance can increase overall plant production and diversity (Connell 1978, Huston 1979), and would have a similar effect on leaf beetles (Şen and Gök 2009). Plants within disturbed vegetation patches are often colonizing species, and so they represent young leaves and less chemical defenses (Wolda 1978, Jolivet 1988, Novotny and Basset 1998) which would favor high abundance of species within some genera of Galerucinae, such as *Centralaphtona* Bechyné & Bechyné, *Chaetocnema* Stephens, *Epitrix* Foudras and *Longitarsus* Berthold. The increase in abundance and species richness as a result of the disturbance of vegetation has been observed in other studies with beetles (Dagobert et al. 2008, Sánchez-Reyes et al. 2012), including Chrysomelidae (Linzmeier and Ribeiro-Costa 2009), because by altering the species composition of a plant community, also alters species composition and abundance of phytophagous beetles (Wąsowska 2004).

On a temporal scale, the Chrysomelidae community followed a markedly seasonal pattern, where the dry season was the most favorable for collection of this group in the study area (greatest abundance and species richness). This is a pattern that was also found in the subtropical region where the higher captures of insects occur in the dry season. Linzmeier and Ribeiro-Costa (2008, 2013) found the major activities of Chrysomelidae during spring/summer, before the rains. Nummelin and Borowiec (1991) in Uganda, also found the peak densities of chrysomelid beetles (subfamily Cassidinae) at the beginning of the dry season after the long rains. Jones et al. (2012) found the greatest richness, diversity and abundance of Apionidae (Curculionoidea) during the dry season in northeastern Mexico. In our study, the greatest number of species and individuals were recorded during the late dry season, resulting in a constant evenness with the increase of the samples; also, the diversity value significantly higher obtained at the early wet season was originated by the high number of species and the drop in abundance value, which can be reflected also in the SHE analysis, where evenness remains constant at the increase of the samples.

Results presented here can be partially explained by the indirect effect of precipitation and temperature on the abundance and species richness, respectively. In this study, abundance increases significantly as precipitation decreases, while species richness increases significantly with an increasing in the maximum temperature. Influence of high temperature present during the wet season has been observed in other studies with Chrysomelidae in tropical areas (Linzmeier and Ribeiro-Costa 2008, 2013). There are several reasons that are related to precipitation and temperature and which can explain the seasonal differences. First, it is probable that many of the leaf beetles are in larval stages during the wet season, and hence lesser abundance of adults was obtained during that season, because we did not collect immatures. At the start of the rainy season, plant density increases which represents greater food availability and new leaf tissues are more palatable for developing immatures (Wolda 1978, Jolivet 1988, Novotny and Basset 1998, Linzmeier and Ribeiro-Costa 2008, 2013). Second, with greater foliage during the wet season, the foliar area in general increases dramatically which results in populations being more “diluted” among the foliage. Thus, the chances of collecting individuals per sweep is reduced because of the great area of foliage. And third, many leaf beetles pass the dry season as adults in high concentrations in microhabitats unrelated to their host plant and vegetation type. There is evidence that beetles will fly away from dry habitats to wetter ones during the dry season, especially to riparian vegetation (Janzen 1973). However, the life-cycles of each species determines the pattern seen in the Chrysomelidae community, and thus an analysis of each species, their host plant and specific behavior needs to be taken in account (Linzmeier and Ribeiro-Costa 2013).

Although our data only considered a single canyon, beta diversity between sites at different altitudes was high. Similarity values obtained for all comparisons of sites were below 50%. Among the sites there was greater similarity of the first two altitudinal sites, with the lower similarity at the higher site. This pattern apparently reflects the greater difference between the tropical and temperate affinities of the vegetation and associated chrysomelids between the lower sites (Neotropical affinities) with the higher site (Nearctic affinities). Similarly, all comparisons between seasons had a similarity less than 50%, and a high number of exclusive species to each season. This pattern of low similarity indicates a significant turnover of species with altitude, which could be caused by the environmental heterogeneity of the area reflected in the different vegetal communities along the altitudinal gradient, which is observed in the three groups formed by the site cluster, and also the three groups (group 1: LWS and EDS; group 2: EWS; group 3: LDS) that were formed in seasonal cluster. However, we also found high values of alpha diversity for each of the sites and analyzed seasons, which suggests that biotic and abiotic factors present at each site and for each station generates unique conditions for certain species of chrysomelid beetles.

Our results highlight the importance of conservation of a heterogeneous habitat which can generate unique species compositions of highly diverse taxa, such as Chrysomelidae. Results presented here establish baseline data for Chrysomelidae richness and diversity for the region and can serve as a reference study for future work on the potential use of Chrysomelidae as indicator group of community diversity in natural areas. However, further studies are still needed to analyze the importance of environmental factors in the distribution and phenology of Chrysomelidae species and possible changes to expect due to climate change.

## Conclusions

A total of 2,226 specimens were collected belonging to six subfamilies, 81 genera and 157 species of Chrysomelidae from the study area. The greatest abundance and density of individuals were recorded at the lowest elevation site; however, alpha diversity increased with increasing altitude, and species richness was higher at intermediate altitude. Similarity values were less than 50% among the three sites indicating that each site had distinct species assemblages of Chrysomelidae.

The highest abundance and species richness was obtained during the late dry season, whereas diversity was higher during the early wet season. Geographical location of the study area plus different vegetal compositions from the three sites sampled could be the principal reason for the variation here found in Chrysomelidae communities with altitude and season. Also, precipitation and temperature may influence the Chrysomelidae community in study area; however, both abiotic factors affect directly the vegetal composition which is assumed to be the principal factor in determining leaf beetle species composition and abundance.

The present work is one of the first specific area studies of Chrysomelidae conducted in Mexico, in which both altitude and season are analyzed. The information presented here provides baseline data that allow for comparisons of the diversity and species richness of Chrysomelidae on a regional and national scale. This information could be used as an initial step to analyze the potential use of Chrysomelidae as an indicator group of biodiversity in Mexico.
